# Fluoroscopy-Guided Transgluteal Pudendal Nerve Block for Pudendal Neuralgia: A Retrospective Case Series

**DOI:** 10.3390/jcm13092636

**Published:** 2024-04-30

**Authors:** Danielle Levin, Daniel Van Florcke, Monika Schmitt, Lucinda Kurzava Kendall, Alopi Patel, Lisa V. Doan, Meera Kirpekar

**Affiliations:** 1 Department of Anesthesiology, Perioperative Care & Pain Medicine, New York University Langone Health, New York, NY 10016, USA; daniel.vanflorcke@nyulangone.org (D.V.F.); lisa.doan@nyulangone.org (L.V.D.);; 2 Department of Physical Medicine and Rehabilitation, New York University Langone Health, New York, NY 10016, USA; monika.schmitt@nyulangone.org (M.S.); lucinda.kendall@nyulangone.org (L.K.K.); 3 Department of Anesthesiology, Critical Care & Pain Medicine, Rutgers-Robert Wood Johnson Medical School, New Brunswick, NJ 08901, USA; patel154@rwjms.rutgers.edu

**Keywords:** pudendal neuralgia, women’s health, pelvic pain, pudendal nerve block

## Abstract

**Background/Objective:** Pudendal neuralgia is a distressing condition that presents with pain in the perineum. While a positive anesthetic pudendal nerve block is one of the essential criteria for diagnosing this condition, this block can also provide a therapeutic effect for those afflicted with pudendal neuralgia. There are multiple ways in which a pudendal nerve block can be performed. The objective of this study is to share our results and follow-up of fluoroscopy-guided transgluteal pudendal nerve blocks. **Methods:** This is a retrospective case series. Included were 101 patients who met four out of the five Nantes criteria (pain in the anatomical territory of the pudendal nerve, pain worsened by sitting, pain that does not wake the patient up at night, and no objective sensory loss on clinical examination) who did not respond to conservative treatment and subsequently underwent a fluoroscopy-guided transgluteal pudendal nerve block. Therapeutic success was defined as a 30% or greater reduction in pain. Success rates were calculated, and the duration over which that success was sustained was recorded. **Results:** For achieving at least 30% relief of pain, using worst-case analysis, the success rate at two weeks was 49.4% (95% CI: 38.5%, 60.3%). In addition to pain relief, patients experienced other therapeutic benefits, such as reductions in medication use and improvements in activities of daily living. **Conclusions:** Fluoroscopy-guided transgluteal pudendal nerve block appears to be effective in patients who have pudendal neuralgia that is resistant to conservative therapy, with good short-term success.

## 1. Introduction

Pudendal neuralgia is a debilitating chronic neuropathic pain condition characterized by perineal pain. It primarily affects women, with an incidence twice as high as in men. Overall, according to the International Pudendal Neuropathy Association, this condition affects 1 in 100,000 individuals [1].

Amarenco et al. [2] first described pudendal neuralgia in 1987 as the “cyclist syndrome”, as the symptoms were first noticed in competitive cyclists. The Nantes criteria are now used for diagnosing pudendal neuralgia: pain in the anatomical territory of the pudendal nerve, pain worsened by sitting, pain that does not wake the patient up at night, no objective sensory loss on clinical examination, and positive anesthetic nerve block [3,4]. A pudendal nerve block, which is part of the diagnostic criteria of pudendal neuralgia, may also serve as a therapeutic treatment.

Many conditions can cause pudendal neuralgia. In females, the three most common causes of pudendal neuralgia are surgery, pelvic trauma, and childbirth. The most common surgeries to cause pudendal neuralgia include repair of a prolapse (vaginal more commonly than rectal), sling procedures, and hysterectomy due to either placement of a mesh or suture that entraps or irritates the pudendal nerve or, possibly, a small blood collection or hematoma compressing the nerve. Most patients that develop post-surgical pudendal neuralgia do so immediately after surgery, but the diagnosis is often missed because the pain is attributed to usual post-operative pain. In a vaginal delivery, the pudendal nerve can become damaged from stretching of the pelvic floor. Other conditions can also cause pudendal neuralgia, such as pelvic floor dysfunction or inflammatory, autoimmune, or infectious conditions like herpes simplex and human immunodeficiency virus infection, diabetes, multiple sclerosis, inflammatory bowel disease, and more [4].

A pudendal nerve block may assist in the diagnosis of pudendal neuralgia, as well as serving as a therapeutic treatment [3]. Various approaches, such as landmark-based, ultrasound-guided, and computed tomography (CT)-guided, have been considered in the past to perform this block via transvaginal, transperineal, or transgluteal needle insertion. However, all of these approaches have some limitations, like high cost, inaccurate/unreliable results, and being difficult or inconvenient to perform in practice. To overcome these limitations, Choi et al., in 2006, described a fluoroscopy-guided approach in the prone position [5]. We performed a retrospective analysis of our experience with fluoroscopy-guided transgluteal pudendal nerve block administration.

## 2. Materials and Methods

### 2.1. Ethical Considerations

This study was conducted according to the guidelines of the Declaration of Helsinki and approved by the Institutional Review Board of NYU Langone Health (protocol #i23-01058; date of approval: 22 September 2023). Patient consent was waived due to this study being a retrospective chart review. All patients included in this study provided written informed consent to receive a fluoroscopy-guided transgluteal pudendal nerve block as part of clinical care.

### 2.2. Study Population

We conducted a chart review of consecutive patients who underwent fluoroscopy-guided transgluteal pudendal nerve block at our single, urban institution from August 2018 to August 2023. Patients aged 18 years and above were included in this retrospective review. All fluoroscopy-guided transgluteal pudendal nerve blocks were directly performed by, or supervised by, the last author of this article. The inclusion criteria were patients who met the following four of the five Nantes criteria for pudendal neuralgia: pain in the anatomical territory of the pudendal nerve, pain worsened by sitting, pain that does not wake the patient up at night, and no objective sensory loss on clinical examination [3]. Patients excluded from this review were the ones who did not meet these four Nantes criteria.

Baseline characteristics, such as age, gender, body mass index, medical comorbidities, and prior treatments, were recorded. Each chart was reviewed by teams of two investigators, each working independently. Any discrepancies were resolved by another independent investigator, who was not involved in the initial chart review.

### 2.3. Pudendal Nerve Block

At the start of each procedural visit, medical history and medications were confirmed with the patient. After re-discussion of the procedure, informed consent was obtained to proceed with the pudendal nerve block.

Each patient was positioned on the fluoroscopy table in the prone position. The patient’s gluteal skin was cleaned with chlorhexidine applicators and draped in a sterile fashion. Sterile technique was adhered to throughout each procedure. Hemodynamics was monitored throughout the procedure with heart rate, pulse oximetry, and blood pressure measurements.

**Figure 1:**
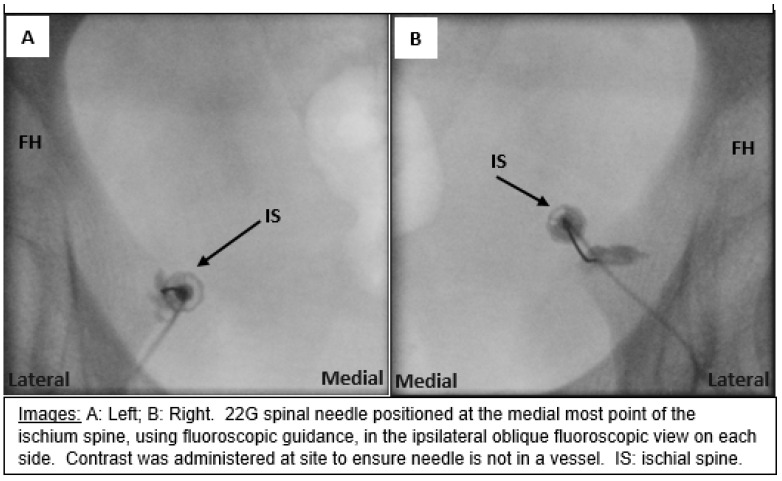
Fluoroscopy-guided images of bilateral pudendal nerve block administration.

Fluoroscopy was positioned with an anteroposterior view, with approximately 15–20° ipsilateral oblique and 0–10°caudad tilts to bring the ischial spine out from behind the superior pubic ramus on the image. The skin was then marked over the ipsilateral spine of the ischium and infiltrated with 1% lidocaine using a 25G needle. A 22G, spinal, 3.5” needle was advanced incrementally under radiographic guidance to the medial-most point of the spine of the ischium. Fluoroscopy views confirmed the placement. The needle was then aspirated, and a small dose of contrast was given to rule out intravenous placement and confirm the needle tip’s location (Figure 1). Injectate consisting of 2.5 mL of 0.25% bupivacaine and 1 mL of 10 mg/mL dexamethasone was injected. The needle was then removed, and a dressing was applied. The procedure was repeated for the other side if the patient’s symptoms were bilateral. The patients were ambulated to the recovery area and monitored for 10–20 min. Upon discharge, the patients were instructed to contact the office immediately if they noticed any swelling, redness, fever, weakness, numbness, and/or discharge. Patients were scheduled for a follow-up appointment two weeks after the procedure.

### 2.4. Outcome Measurements

For this study, outcomes were measured after a single pudendal nerve block procedure. Patients were asked to report the percentage of pain reduction that they experienced at follow-up visits. If a patient had any pain relief from the procedure, the patient met the fifth requirement of the Nantes criteria, signifying that the patient met the full diagnostic criteria for pudendal neuralgia. Changes in the ability to conduct activities of daily living, changes in medication use, and post-procedure complications were noted.

Data were analyzed using Microsoft Excel. Data were presented as the mean ± standard deviation for continuous variables and as numbers and percentages for categorical variables. Therapeutic success of fluoroscopy-guided transgluteal pudendal nerve block was defined as at least a 30% reduction in pain [6,7]. Success rates were calculated, and the duration over which success was sustained was recorded. As there was variability in the number and duration of follow-up periods, of the patients who reported a positive response to the pudendal block, a worst-case analysis was used to assess the duration of pain relief.

## 3. Results

### 3.1. Participant Flow

Of the 101 patients who received a fluoroscopy-guided transgluteal pudendal nerve block between August 2018 and August 2023 at our institution, 5 patients were excluded from the analysis as they had no follow-up data. Fifteen patients did not receive any pain relief from the block, so they did not meet the fifth Nantes criterion for pudendal neuralgia; imaging was reviewed to ensure that the needle placement was appropriate to achieve a pudendal nerve block. Out of the 81 remaining patients who experienced pain relief after the block and, thus, met the fifth Nantes criterion for pudendal neuralgia, 63 (77.8%) were female and 18 (22.2%) were male, with a mean (± standard deviation) age of 43.6 ± 16.1 years and BMI of 29.2 ± 12.9 kg/m^2^. The average baseline pain at the moment of the initial office visit was 4.75 ± 2.6 out of 10 on the numeric rating scale. The most common condition of past medical history was a history of gynecological or urological surgery (60.4%) (Table 1).

### 3.2. Primary Outcomes

**Figure 2:**
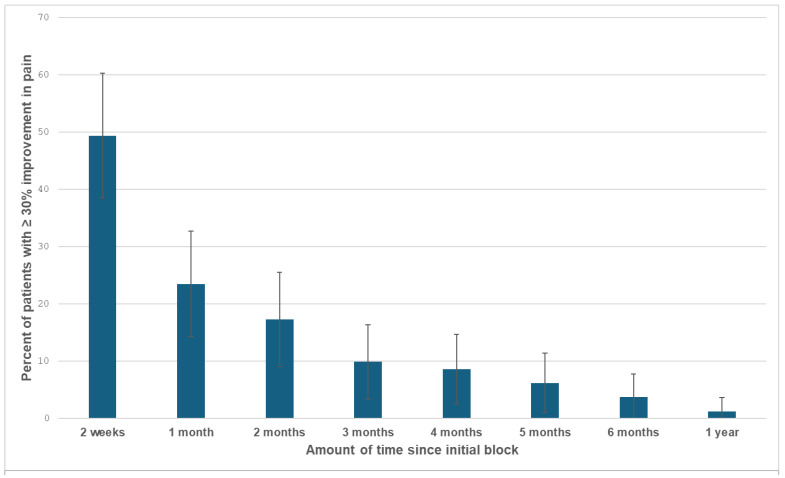
The success rates (≥30% improvement in pain) at each timepoint. The error bars are the 95% confidence intervals.

The success rates (≥30% reduction in pain) were 49.4 (95% CI: 38.5%, 60.3%) lasting at least two weeks and 23.5% (95% CI: 14.3%, 32.7%) lasting at least one month. The relief persisted for various periods (Figure 2), ranging from one day to over a year.

### 3.3. Secondary Outcomes

In addition to pain relief, some patients reported that they noticed changes in their daily life after receiving the fluoroscopy-guided transgluteal pudendal nerve block. Patients noted a reduction in their pain medication use, headaches, and menstrual cramping. Others commented on their improved ability to wear tight clothing, sit on hard surfaces, exercise, engage in vaginal sexual intercourse, or use dilators/tampons, as well as reduced bowel/bladder symptoms. A total of 14 out of 81 patients (17.3%) reported some type of adverse effect from the block, with the most common symptom reported being temporary pain flare-up and temporary leg weakness/numbness. All of the complications were temporary and resolved within several hours, except for one patient who reported a sensation of numbness in the leg for four days (Table 2).

## 4. Discussion

### 4.1. Our Retrospective Case Series

To the best of the authors’ knowledge, our study is the largest retrospective case series to evaluate the effectiveness of fluoroscopy-guided transgluteal pudendal nerve blocks. All of these procedures were covered by insurance.

In our study, we noted short-term pain relief, with only a few temporary adverse events, after pudendal nerve block under fluoroscopic guidance. In addition to pain relief, a number of our patients also experienced improvement in their activities of daily living after the block. Based on these findings, it seems that a transgluteal pudendal nerve block under fluoroscopic guidance could be a useful treatment option for those afflicted with pudendal neuralgia. However, many other treatment options for the management of pudendal neuralgia can be considered as well.

### 4.2. Non-Interventional Treatment Options

Currently, limited medical literature is available on the diagnosis and treatment of pudendal neuralgia [2,4,8,9]. However, different treatment options do exist for the management of this condition, including both non-interventional and interventional approaches. Non-interventional options include lifestyle modifications, physical therapy, cognitive behavioral therapy, medications, and alternative therapies.

#### 4.2.1. Lifestyle Modifications

Initial lifestyle modifications can help protect the pudendal nerve from aggravation of the neuropathy. For instance, since pain occurs while seated, a simple “sit-pad” or “doughnut” can be purchased commercially, or self-constructed, and used when the patient is sitting. Likewise, other lifestyle modifications are seldom considered in modulating chronic pain but are increasingly drawing attention for improving both health and pain. The six tenets of lifestyle medicine include adopting a whole-food diet (predominantly plant-based), improving sleep, optimizing physical activity, managing stress with healthy coping strategies, forming and maintaining positive relationships, and ceasing tobacco and minimizing alcohol and opioid use. Ultimately, the concept is inflammation—poor lifestyle choices lead to increased inflammation in the body, which worsens pain [10,11].

In terms of food, an unhealthy diet high in processed foods, saturated fats, red meat, high-fat dairy products, and high-sugar foods can alter the body’s gut microbiome and immune system, increasing inflammation in the body—particularly markers of inflammation such as C-reactive protein, certain interleukins, and tumor necrosis factor alpha—worsening chronic pain conditions. Studies have shown evidence of this with regard to chronic pain conditions like fibromyalgia, chronic fatigue syndrome, inflammatory bowel disease, and more, and this can be extrapolated to pain conditions such as pudendal neuralgia. Incorporating a whole-food diet can decrease inflammation and improve pain, mood, and overall health [10,11].

Poor sleep can have similar effects. Roughly two-thirds of chronic pain patients also suffer from poor sleep, and women have a 40% higher risk of insomnia than men. Studies have shown that ideal sleep for most adults lies in the range of 7–9 h of sleep per night, or 3–4 full REM cycles, and for every one hour less of sleep per night one gets, inflammatory mediators increase by 9–12% in the body, thereby worsening pain. Restorative sleep has been shown to improve pain as well as depression [10,11].

Regular physical activity also acts as an anti-inflammatory therapy, as loss of fat and increased muscle production lead to decreases in TNF-alpha and increases in IL-6 (an anti-inflammatory cytokine), leading to decreases in pain. Studies have shown physical activity to be particularly helpful in arthritis, fibromyalgia, chronic fatigue syndrome, and more, and it could be helpful for pudendal neuralgia as well. Increased levels of stress lead to more cortisol release, increased adrenalin, and decreased immune function, leading to more pro-inflammatory cytokines being released in a “fight or flight” response. Managing stress leads to reductions in pro-inflammatory cytokines, thereby reducing pain. Positive psychology leads to reductions in levels of anxiety and depression; anxiety and depression are conditions that are known to exacerbate chronic pain, so maintaining positive connections can lead to improvements in pain perception [10,11].

Toxic substances such as tobacco, alcohol, and potentially opioids can also worsen pain due to increases in inflammatory mediators. Alcohol, nicotine, and opioids can contribute to poor sleep due to a decrease in sleep quality with suppression of REM sleep, thereby also increasing inflammation and worsening pain [10,11].

#### 4.2.2. Physical Therapy

Pelvic floor physical therapy focuses on either strengthening or relaxing the pelvic floor muscles to relieve pressure on the bones, organs, and nerves. It helps with sexual dysfunction, pelvic pain, urinary symptoms, constipation, inflammatory bone conditions, and more. The therapy can include Kegel exercises, but it is important to note that in some cases this can worsen pelvic pain by increasing pressure, so a physical therapist will begin by first assessing the muscles to determine which muscles need what type of exercise. This can be achieved via biofeedback, which uses a device to check the contraction of pelvic floor muscles in order to assess how the exercises are working and what areas require improvement. Pelvic floor physical therapy can also include electrical stimulation, including a TENS (transcutaneous electrical stimulation) unite. A TENS unit may also be helpful to stimulate the nerve roots at the sacral level or the perineal level [12,13].

#### 4.2.3. Cognitive Behavioral Therapy

Cognitive behavioral therapy can be very helpful according to multiple studies. It focuses on anxiety, depression, and preventing and/or treating catastrophizing behavior to reduce pain and improve sexual function [14,15].

#### 4.2.4. Pharmacological Therapy

Medications can also be helpful, particularly in the classes of muscle relaxants, anticonvulsants, and antidepressants. Muscle relaxants, such as methocarbamol, cyclobenzaprine, tizanidine, and baclofen, help to relax the pelvic floor muscles to relieve pressure on the nerves. Anticonvulsants and antidepressants work on the nerves peripherally to treat neuropathic pain and act centrally to reduce central sensitization and to treat allodynia and hyperalgesia. Anticonvulsants used for pain include gabapentin, pregabalin, and topiramate. Antidepressants used for pain include tricyclic antidepressants, such as amitriptyline and nortriptyline, and serotonin–norepinephrine reuptake inhibitors, such as duloxetine and venlafaxine.

Also, recently, it has been reported that low-dose naltrexone can help manage different types of chronic pain. Naltrexone, an oral opioid antagonist used to treat substance use disorder, when used at an off-label dose of 0.1–5 mg, has been used to treat various chronic conditions. The analgesic mechanism of low-dose naltrexone is unclear, but some have suggested that there are anti-inflammatory properties related to LDN’s Toll-like receptor 4 antagonism [16,17]. Brown et al. found that low-dose naltrexone works best to manage chronic neuropathic pain [18]. Extrapolating from that, low-dose naltrexone could be quite helpful for pudendal neuralgia as well, given that pudendal neuralgia is a neuropathic condition.

### 4.3. Interventional Treatment Options

Although a multitude of non-interventional treatment options exist for pudendal neuralgia, based on the Nantes criteria, pudendal neuralgia cannot actually be diagnosed without a pudendal nerve block.

#### 4.3.1. Nerve Block

A pudendal nerve block can be diagnostic as well as therapeutic. The injection can be performed with local anesthetic only, or with local anesthetic plus a steroid. The steroid component is meant to make the effects of the injection last longer, but the data are mixed as to whether the steroid prolongs the effects of the block. More data, however, suggest the use of a steroid to prolong the effect of the block, and the effects last at least 3–6 months on average [3,19].

The pudendal nerve block can be performed either “blind” or image-guided using ultrasound, fluoroscopy, or computed tomography. Obstetricians/gynecologists typically perform the injection transvaginally, at Alcock’s canal. Pain physicians, on the other hand, typically perform the injection transgluteally, at the ischial spine. For a transgluteal injection, imaging is strongly recommended for better visualization and a more precise injection.

A randomized, single-blind, split-plot design was used to compare ultrasound-guided transgluteal pudendal nerve blocks to fluoroscopy-guided pudendal nerve blocks. No differences in the degree of neural blockade or adverse effects were found between the two treatment methods. However, the time to complete the block was significantly longer with ultrasound guidance [20]. On the other hand, when fluoroscopy is used, the patient and the healthcare providers are exposed to radiation.

To date, there have been only two case reports in the literature about the therapeutic effectiveness of fluoroscopy-guided transgluteal pudendal nerve blocks [21,22]. Yu et al. described a patient with severe intractable vulvar pain after bladder cancer surgery and adjuvant radiation therapy, who received five fluoroscopy-guided pudendal nerve blocks, which cumulatively resulted in a reduction in her pain. From an initial numeric rating scale of ten, the patient’s pain became a three, and the patient was able to further manage her pain with oral medication [21]. Similarly, Lee et al. described a patient with chronic pelvic and perineal pain who underwent a fluoroscopy-guided pudendal nerve block and experienced pain reduction from an initial numeric rating of nine to six, which lasted for two weeks [22].

In our study, we saw short-term improvements in pain after pudendal nerve block under fluoroscopic guidance. Our patients also experienced improvement in their activities of daily living after the block. Therefore, transgluteal pudendal nerve block under fluoroscopic guidance could be a useful, precise treatment option for pudendal neuralgia.

If the first pudendal nerve block provides only diagnostic information but does not provide a therapeutic element (pain relief lasting at least a few weeks/months), other procedures can be pursued to provide prolonged pain relief. One option is to repeat the pudendal block, and sometimes the repetition of the block provides longer pain relief. We currently do this at our clinic, and we plan on evaluating its effectiveness in a future manuscript.

#### 4.3.2. Radiofrequency Ablation

Another option for durable pain control is pulsed radiofrequency ablation. Studies have used pulsed radiofrequency ablation under computed tomography guidance (although the radiofrequency ablation can also be carried out under fluoroscopy or US guidance), but the studies on this technique are very few in number, and more information is still needed [23,24]. At our clinic, some of our patients have received pulsed radiofrequency ablation under fluoroscopic guidance, and their data will also be reported in a future manuscript.

#### 4.3.3. Neuromodulation

Pudendal neuromodulation can also be considered. There are currently three different kinds of stimulators available on the market—traditional stimulators, dorsal root ganglion stimulators, and peripheral nerve stimulators. Traditional spinal cord stimulators consist of thin wires, called electrodes, and a small battery pack, the generator. The electrodes are inserted into the epidural space, and the generator is placed under the skin, usually near the abdomen or buttocks. Patients use a remote control to send electrical impulses when they feel pain. This mechanism can target multiple muscle groups directly from the spine and may even alter how the brain perceives pain. Traditional spinal cord stimulators provide patients with a light tingling sensation, paresthesia, in place of the sensation of pain.

Dorsal root ganglion stimulators, in contrast to traditional spinal cord stimulators, are much more specific to the affected body part. These devices target the densely populated sensory nerves, which regulate sensations and signals that travel through nerve fibers along the spinal column to the brain. Since the spinal column has many different dorsal root ganglions, each of which is associated with different areas of the body, the dorsal root ganglion stimulator can target the sacral nerve specifically [25]. A retrospective review by Schu et al. investigated the use of dorsal root ganglion stimulation in the management of groin pain and found that, at follow-up (an average of 27.8 weeks), 82.6% of patients experienced a >50% reduction in pain [26]. Hunter et al. conducted a case series of dorsal root ganglion stimulation in seven patients with chronic pelvic pain who had leads placed over the bilateral L1 and S2 dorsal root ganglions, finding that all of the patients experienced significant pain relief [27]. Although clinical randomized control trials on dorsal root ganglion stimulation are sparse, it appears that dorsal root ganglion stimulation may be an effective treatment for neuropathic groin pain.

Peripheral nerve stimulation involves surgery that places an electrode next to one of the peripheral nerves (nerves that are located beyond the spinal cord and brain). The peripheral nerve stimulator electrode delivers rapid electrical pulses, which cause patients to feel paresthesia. The patients are able to control the stimulation by adjusting the stimulation parameters as needed. Several case series have been conducted with sacral root stimulation, which demonstrated that this could be a promising option for managing pelvic pain as well [28,29,30,31,32].

However, few studies have reported specifically on pudendal nerve stimulator treatments, but since neuromodulation is quite successful for other chronic nerve conditions, it can be considered for pudendal neuralgia as well [25,26]. This must be balanced against the potentially prohibitive costs to the patient due to difficulty with insurance coverage.

#### 4.3.4. Surgery

Surgery can be considered as well, in the form of pudendal nerve release surgery. There are multiple surgical approaches suggested, including transgluteal, transperineal, laparoscopic, and gluteal endoscopic approaches, with no particular approach showing superiority over others. However, surgeons who perform this procedure are uncommon, and the costs of the procedure can be difficult to manage due to insurance coverage. Overall, surgery has been shown to about 60–80% effective when patients show all five of the Nantes criteria discussed earlier [33].

## 5. Limitations of the Study

As far as the authors are aware, our study is the largest retrospective case series to evaluate the effectiveness of fluoroscopy-guided transgluteal pudendal nerve block for the treatment of pudendal neuralgia. However, the biggest limitation of this study is its small sample size. One reason for the small sample size was loss to follow-up.

Also, it is difficult to interpret the pre-procedure pain numeric rating scale, since pain with pudendal neuralgia is not always present and is more likely to present with certain specific activities during the day. Also, when the numeric rating scale question was asked, it was not specified whether that number was based on the pelvic pain or another pain area of the patient, as some patients presented to the clinic with multiple pain concerns.

Another limitation is external validity, insofar as the method by which pudendal nerve blocks are performed varies widely in the pain medicine community. In our study, the pudendal block was performed in a uniform manner, but other institutions may be performing this block differently. Moreover, our study did not control for the use of concurrent medications. Therefore, our pudendal nerve block results may not be applicable to other block approaches or in patients with concurrent medication use.

## 6. Conclusions

In this retrospective review, we found that a fluoroscopy-guided transgluteal pudendal nerve block offered clinically important short-term benefits for pudendal neuralgia. The procedure was well tolerated, with few adverse events, all of which were transient in nature.

## Figures and Tables

**Table 1:** Patient characteristics.

Baseline Characteristics	Mean ± Standard Deviation
Age (years)	43.6 ± 16.1
BMI (kg/m^2^)	29.2 ± 12.9
Gender (Female)	63 (77.8)
Gender (Male)	18 (22.2)
Past medical history	N (%)
Anxiety	29 (35.8)
Depression	23 (28.4)
Autoimmune conditions	28 (34.6)
Inflammatory bowel disease	11 (13.6)
Gynecological/urological surgery	49 (60.4)
Traumatic birth	5 (5.2)
Prior Treatments	N (%)
Lifestyle modifications	18 (22.2)
Psychotherapy	1 (1.2)
Acupuncture	8 (9.9)
Pelvic floor physical therapy	57 (70.4)
Pharmacological medications	74 (91.4)
Other procedures/surgeries	49 (60.5)

Continuous variables are expressed as the mean ± standard deviation. Categorical variables are expressed as number (percentage).

**Table 2:** Complications associated with fluoroscopy-guided transgluteal pudendal nerve block.

Complications	N (%)
Fecal incontinence on day of procedure	1 (1.2)
Leg weakness/numbness	7 (8.6)
Pelvic floor spasm	1 (1.2)
Pain flare-up	6 (7.4)
Back pain	1 (1.2)

Categorical variables are expressed as number (percentage).

## Data Availability

The raw data supporting the conclusions of this article will be made available by the authors on request.
